# Repeatability of retinal vessel flicker responses in healthy individuals

**DOI:** 10.1111/aos.17578

**Published:** 2025-08-12

**Authors:** Angelos Kalitzeos, Robert J. Summers, Rebekka Heitmar

**Affiliations:** ^1^ UCL Institute of Ophthalmology University College London London UK; ^2^ Independent Researcher Derby UK; ^3^ University of Huddersfield School of Applied Sciences, Centre for Vision across the Life Span (CVLS) Huddersfield UK

**Keywords:** flicker provocation, neurovascular coupling, repeatability, retinal vessel analyser

## Abstract

**Purpose:**

To determine the repeatability of retinal blood vessel parameters (from proprietary software and the raw vessel data) measured in vivo in healthy individuals at rest, during and post flicker light (FL) provocation using a standardised protocol.

**Methods:**

We recorded the diameters of one retinal artery and one vein in each of 33 healthy adult volunteers at rest, during and post FL provocation on two occasions using the Retinal Vessel Analyser (RVA). Repeat visits were scheduled at three different timepoints: (1) within 30 mins on the same day, (2) within a fortnight and (3) within a month (*n* = 11, each). All participants underwent intraocular and systemic blood pressure assessment to ensure these were comparable between visits.

**Results:**

Retinal vessel parameters at rest, during and post FL provocation were comparable between all pairs of visits for all three groups. Repeatability between visits was assessed using Bland–Altman Analyses and Intraclass Correlation Coefficients (ICCs). Maximum dilation for arteries and veins and maximum constriction for arteries due to FL provocation computed from raw dilation data showed better repeatability than that generated by the RVA software. Time to reach maximum dilation in arteries and veins and maximum constriction in arteries was on average comparable but least repeatable between visits.

**Conclusions:**

Retinal vessel parameters computed from the raw RVA data may be superior in quality to the output from the proprietary software because the latter uses fixed narrow time‐windows to determine the parameters. Variance within healthy controls, pathology groups and repeatability parameters alongside systemic haemodynamic parameters should be considered when utilising dynamic retinal vascular parameters as study endpoints.

## 1 INTRODUCTION

When introduced, the Retinal Vessel Analyser (RVA, Imedos, Germany) was used to record retinal vessel diameters across time, with high temporal and spatial resolution but under constant illumination (baseline conditions) only (Pache et al., 2002; Polak et al., 2000). Until then, quantification of retinal diameters relied heavily on static retinal photography. With the introduction of flicker light stimulation, which induces increased metabolic demand in the retinal tissue and the capability to measure in vivo retinal vascular reactivity, a plethora of different measurement protocols and parameters have been introduced (Heitmar & Summers, 2012; Streese et al., 2021).

Despite a substantial number of studies detailing attempts to exploit the potential of RVA for risk prediction and stratification in a number of pathologies, there has been limited work in examining the repeatability of flicker responses. Studies reporting on the repeatability of the flicker responses are either irrelevant to the current hardware and protocol or incomplete. Polak et al. reported a 25% Coefficient of Variation (CV) of flicker‐evoked retinal diameter responses (*n* = 9) across 5 repeats, but it is unclear whether this reflected arterial, venular or combined data (Polak et al., 2002). A later study (Garhöfer et al., 2003) reported flicker‐induced vasodilation CV values (*n* = 12) of 15.2% for arteries and 20.3% for veins, across two repeats. However, both aforementioned studies used an early model of the RVA system (spectrally separated illumination and flickering light), a flicker frequency of 8 Hz (unlike the current 12.5 Hz) and a protocol of 60 s of baseline illumination followed by 60 s of flickering light. Nagel et al. (2006) were the first to report on short‐term (1 h) and long‐term (1 month) CV values of flicker responses (~1%) using the current measurement protocol but an older camera model (*n* = 28) and measured only one parameter: Maximum Dilation (MD) of arteries and veins averaged across three flicker cycles. Short‐term (30–60 min) repeatability of values calculated with the inbuilt RVA analysis has been reported in healthy Asians (*n* = 33) using the Pearson correlation coefficient and Bland–Altman (B‐A) analyses (Nguyen et al., 2009), while correlation analyses is a poor tool to evaluate repeatability (Halligan, 2002) as it only shows the strength of linear association between two variables and lacks information on measurement agreement; B‐A analyses is a more suitable method to assess measurement agreement (Giavarina, 2015). While their Pearson correlation coefficients for baseline diameters and maximum dilation for arteries and veins were 0.95 (arteries) and 0.98 (veins), 0.85 (arteries) and 0.8 (veins), respectively. The B‐A analyses, however, showed a bias of 0.044 for arterial diameters and 0.217 for venous diameters due to flicker provocation with 95% limits of agreement (LOA) for retinal arterioles and venules of 2.060/−1.971 and 8.847/−2.414, respectively. A later publication (*n* = 78) on the repeatability of individual flicker responses (per flicker cycle) reports data on CV values for MD (arteries: 1.3 ± 1.1%; veins: 1 ± 0.8%), Maximum Constriction (MC) (arteries: 1.2 ± 0.9%; veins: 0.8 ± 0.6%) and Reaction Time (RT) (arteries: 31.5 ± 19.3%; veins: 17.9 ± 12.7%) (Heitmar et al., 2010). Short‐term retesting (30 min) has also been examined in healthy participants (*n* = 20) with intraclass correlation coefficient (ICC) values of 0.94 and 0.56 for arterial and venular MD, respectively (Noonan et al., 2013).

Despite a feature review on the RVA and its applicability, highlighting several ‘unresolved open questions’ (Garhöfer et al., 2010) in particular reproducibility of flicker responses, few have been addressed to date. The aim of this study was to evaluate the repeatability of vascular parameters (for both arteries and veins) as measured with the standard 12.5 Hz, three cycle flicker protocol, in healthy participants.

## 2 METHODS

### 2.1 Participants

All measurements were performed by a single examiner (AK). Tests were performed twice in one arbitrarily selected eye in 33 healthy volunteers. Repeat tests took place within (a) 15–30 mins (short‐term), (b) 3–14 days (mid‐term) and (c) 16–28 days (long‐term) with 11 individuals in each group, respectively. Mid‐term repeat tests took place at the same time of the day, ranging from 0 to 52 mins apart. Long‐term repeat tests took place at the same time of the day for the majority (*n* = 7), ranging from 1 to 60 mins apart, with the remaining 4 participants tested between 3.5 and 8.5 h apart. All participants conformed to the following criteria: aged over 18 years, with no history of systemic or ocular vascular disease or epilepsy, medication‐free and with clear optical media. For a minimum of 12 h prior to testing, participants were instructed to abstain from smoking, consuming alcoholic or caffeinated products, as well as from taking up any sort of strenuous physical activity.

Written informed consent was obtained from all participants. The study complied with the tenets of the Declaration of Helsinki, and ethics approval was granted by the Aston University Ethics Committee.

### 2.2 Data acquisition

All measurements took place in a temperature‐controlled room (21–24°C). Participants remained seated for at least 20 min prior to the start of the examination to ensure stable haemodynamic conditions. Non‐contact tonometry (Pulsair EasyEye, Keeler Ltd., UK) was performed to assess intraocular pressure (IOP) using a validated method and protocol (Ogbuehi & Almubrad, 2008). Three readings were obtained from each eye, and the average value was recorded.

Blood pressure (BP) was measured from the brachial artery of the left forearm using a validated (Rogoza et al., 2000), automated, oscillometric, digital BP monitor (UA‐767, A&D Instruments Ltd., UK). Three consecutive readings of Systolic BP (SBP), Diastolic BP (DBP) and Heart Rate (HR) were recorded, and mean arterial BP (MABP) was calculated according to the formula:

(1) $$\text{MABP}=\frac{2}{3}\mathrm{DBP}+\frac{1}{3}\mathrm{SBP}$$

**FIGURE 1:**
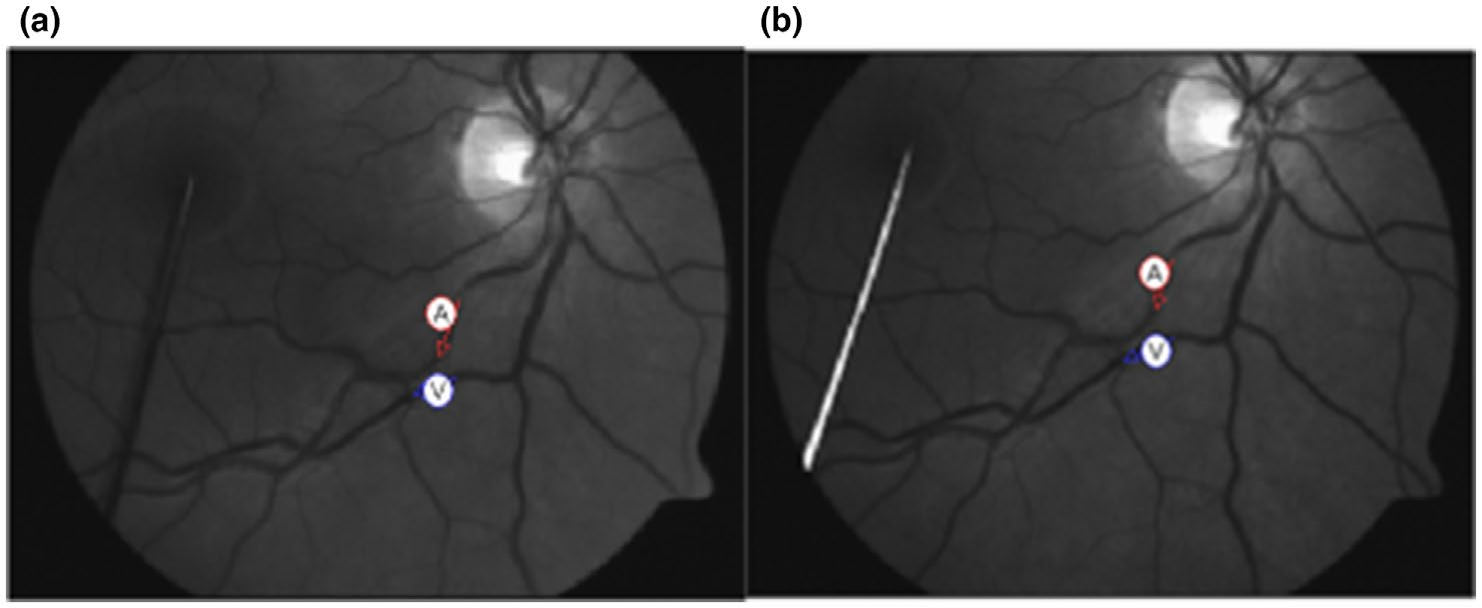
Sample vessel selection. (a) First measurement session, (b) Second measurement session. Red arrow denotes the arterial segment and blue arrow the venular one.

Details on the measuring principle of the RVA have been described previously (Nagel et al., 2004). The standard flicker protocol (Nagel et al., 2005) was carried out, which comprises 50 s of baseline illumination (of which the initial 20 s were consequently discarded from analysis), followed by three 100‐s cycles of 20 s of flickering light and 80 s of recovery. Following full pupil dilation using Tropicamide (1% w/v, Bausch & Lomb, UK), dynamic retinal vessel assessment in one arbitrarily selected eye commenced. The fellow eye was covered to achieve good fixation. During the examination, subjects were encouraged to blink normally (to maintain a sufficiently wet cornea) and to keep steady fixation. A pin that served as a fixation target was placed accordingly in order to position the desired vessel segments centrally as viewed on the computer monitor (Figure 1). The measurement location selected was a minimum of 1 disc diameter (DD) away from the rim of the optic nerve head (ONH). First, the arterial vessel segment was selected, then the venular one, and as soon as the software registered successfully the corresponding positions, the measurement session started automatically. In case the contrast between the vessels and the background retinal tissue was inadequate, or other degradations affecting video quality appeared, the measurement was aborted and restarted.

### 2.3 Standardisations

Each participant's inferior temporal vessels were assessed. When the patient is in slight upward gaze with a dilated pupil, this ensured that the upper lid was naturally positioned higher than in straight forward or slight down gaze. The higher lid position meant it was less likely to reduce the pupillary aperture; therefore, ensuring a more consistent and uniform illumination throughout the measurement cycle.

Vessel length selection was governed by each individual's angioarchitecture but was always kept as long as possible. Nevertheless, when selecting vessel segments for a measurement in real time, no two pairs can be selected to be precisely of equal length. Thus, in order to standardise comparisons and to eliminate potential confounding effects of the segment length measured across repeats, the following procedure was applied: for each vessel pair, measured segments were truncated to exactly equal lengths by processing the raw data matrix output of the RVA. Moreover, for the repeated measurement, the repetition feature of the software was used, which automatically measures at exactly the same location as in the initial measurement session. In a few cases, this was not possible due to registration issues; then the location was manually matched as closely as possible.

### 2.4 Outcome variables tested

**FIGURE 2:**
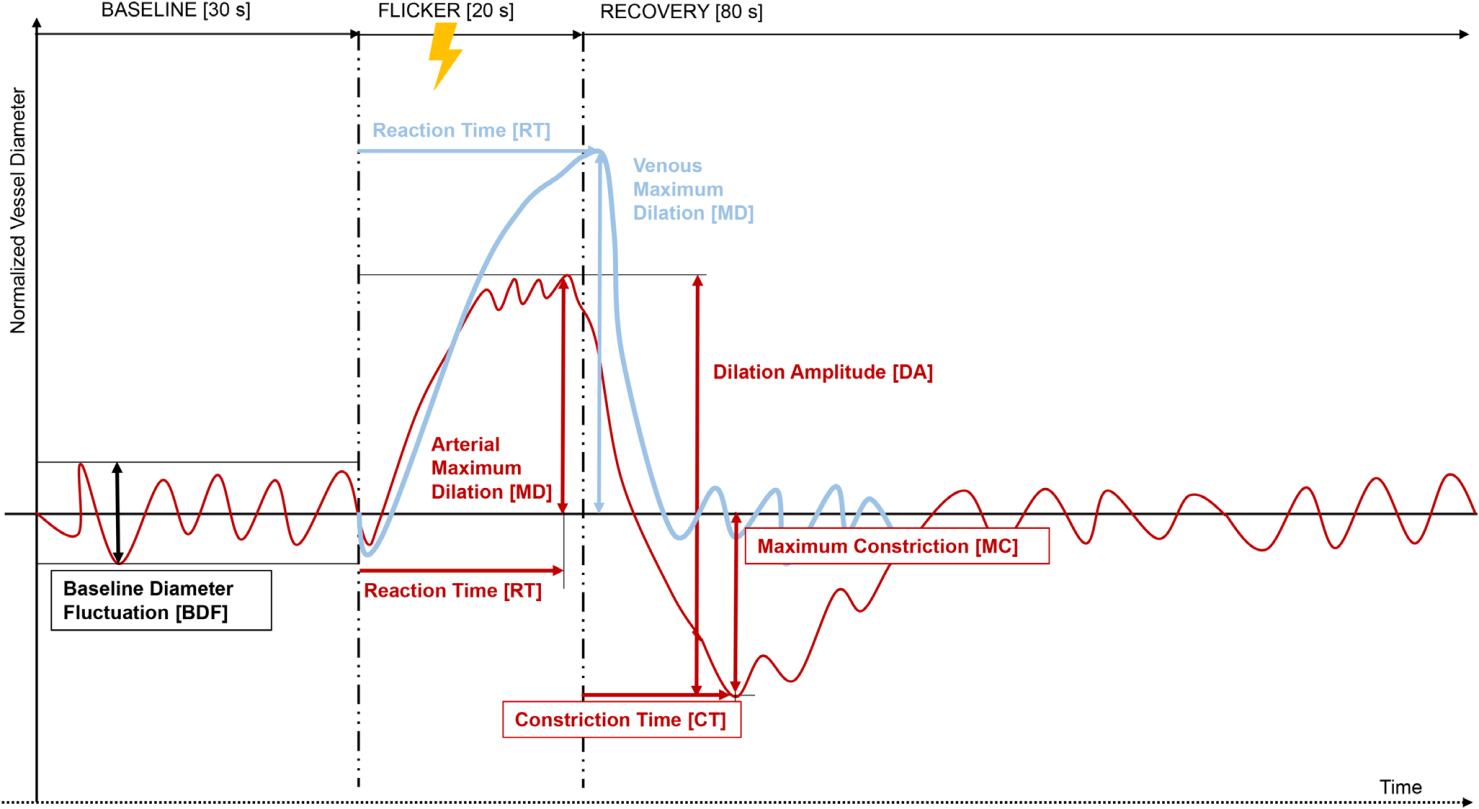
Schematic showing the calculation of retinal vessel parameters from raw Retinal Vessel Analyser data. Parameters in red were calculated for arteries, parameters in blue were calculated for veins and parameters in black were calculated for both arteries and veins.

Data were analysed independently of the vendor‐supplied software. Custom‐built scripts were implemented to process raw RVA data. Details are provided elsewhere (Kalitzeos, 2014). The following parameters were calculated (a schematic can be seen in Figure 2) and compared across the two measurement sessions, averaging all three flicker cycles per session:

- Baseline diameter fluctuation (BDF), the maximum amplitude (peak‐to‐peak) for arteries and veins, 30 s prior to (each) flicker start (Nagel et al., 2004)
- Maximum dilation (MD), the maximum 1‐s diameter for arteries and veins, within 50 s after (each) flicker start (Heitmar et al., 2010)
- Maximum constriction (MC), the minimum 1‐s diameter for arteries, within 50 s after (each) flicker start (Heitmar et al., 2010)
- Dilation amplitude (DA), the difference between MD and MC for arteries (Heitmar et al., 2010)
- Baseline‐corrected flicker response (bFR), the difference between DA and BDF for arteries (Nagel et al., 2004)
- Reaction time (RT), time (in seconds) to reach MD for arteries and veins (Heitmar et al., 2010)
- Constriction time (CT), time (in seconds) to reach MC for arteries (Heitmar, Blann, et al., 2011; Heitmar, Cubbidge, et al., 2011)

Arteriolar and venular diameters relative to the baseline were recorded in measuring units (MU) which correspond to μm in the Gullstrand model eye and compared across repeats. In addition, we report the inbuilt RVA software‐generated parameters of *A*_max_, *A*_min_, *A*_peak_ and *V*_max_ and compare these to arterial MD, MC, DA and venular MD, respectively. Software‐generated dilatory responses, for arteries (*A*_max_, *A*_min_, *A*_peak_) and veins (*V*_max_) represent the averaged responses across 3 flicker cycles, also averaged within an arbitrarily chosen time window that encompasses 6 s: 3 s before and 3 s after flicker cessation (Mandecka et al., 2007).

### 2.5 Statistical analysis

SPSS (Version 13.0, Chicago, SPSS Inc.) was used for all statistical analyses. Graphs were created in R (version 4.4.3). Normality tests were performed on all continuous data by means of the Shapiro–Wilk test to determine distribution. Where normally distributed, data are expressed as means ± SD and repetitions are compared by paired‐samples *t*‐tests. Group means were compared with one‐way analysis of variance. Outcome variables were compared between measurement sessions using Bland–Altman plots. Test–retest repeatability was assessed by calculating ICC values (single measures) with a 95% confidence interval (CI) (two‐way mixed effects model, absolute agreement) (Koo & Li, 2016). For all statistical tests, an alpha level of 0.05 was used.

## 3 RESULTS

Participant characteristics for each measurement session (M1, M2) and for all three repeat subgroups – (a) short‐term, (b) mid‐term and (c) long‐term – are shown in Table 1. A representative retinal arterial and venular recording of one individual across two measurement sessions 7 days apart (mid‐term) is shown in Figure 4.

**TABLE 1:** Participants' characteristics across (a) short‐term (15–30 mins), (b) mid‐term (3–14 days) and (c) long‐term (16–28 days) repeat measurements (M1, M2). Values are expressed as means ± SD. MU stands for Measurement Units corresponding to μm for the Gullstrand model eye.

	M1	M2	M1	M2	M1	M2
Characteristic	a (*n* = 11)		b (*n* = 11)		c (*n* = 11)	
Age (years)	32 ± 9		35 ± 10		28 ± 5	
Males: Females	5: 6		7: 4		3: 8	
Caucasian: Asian	8: 3		9: 2		5: 6	
Intraocular pressure (mmHg)	13 ± 3	12 ± 3	12 ± 4	12 ± 4	13 ± 4	12 ± 3
Systolic blood pressure (mmHg)	110 ± 17	111 ± 16	114 ± 10	111 ± 14	117 ± 14	115 ± 14
Diastolic blood pressure (mmHg)	68 ± 13	69 ± 13	73 ± 8	72 ± 6	72 ± 11	72 ± 9
Mean arterial blood pressure (mmHg)	82 ± 14	83 ± 13	87 ± 8	85 ± 7	87 ± 12	87 ± 10
Heart rate (pulses/min)	72 ± 8	71 ± 9	69 ± 7	69 ± 8	71 ± 12	73 ± 12
Artery diameter (MU)^a^	118 ± 17	119 ± 17	118 ± 23	118 ± 23	120 ± 10	119 ± 10
Vein diameter (MU)^a^	146 ± 21	144 ± 21	150 ± 20	148 ± 18	155 ± 15	156 ± 15

^a^ Absolute baseline blood vessel diameter values provided by the retinal vessel analyser.

**FIGURE 3:**
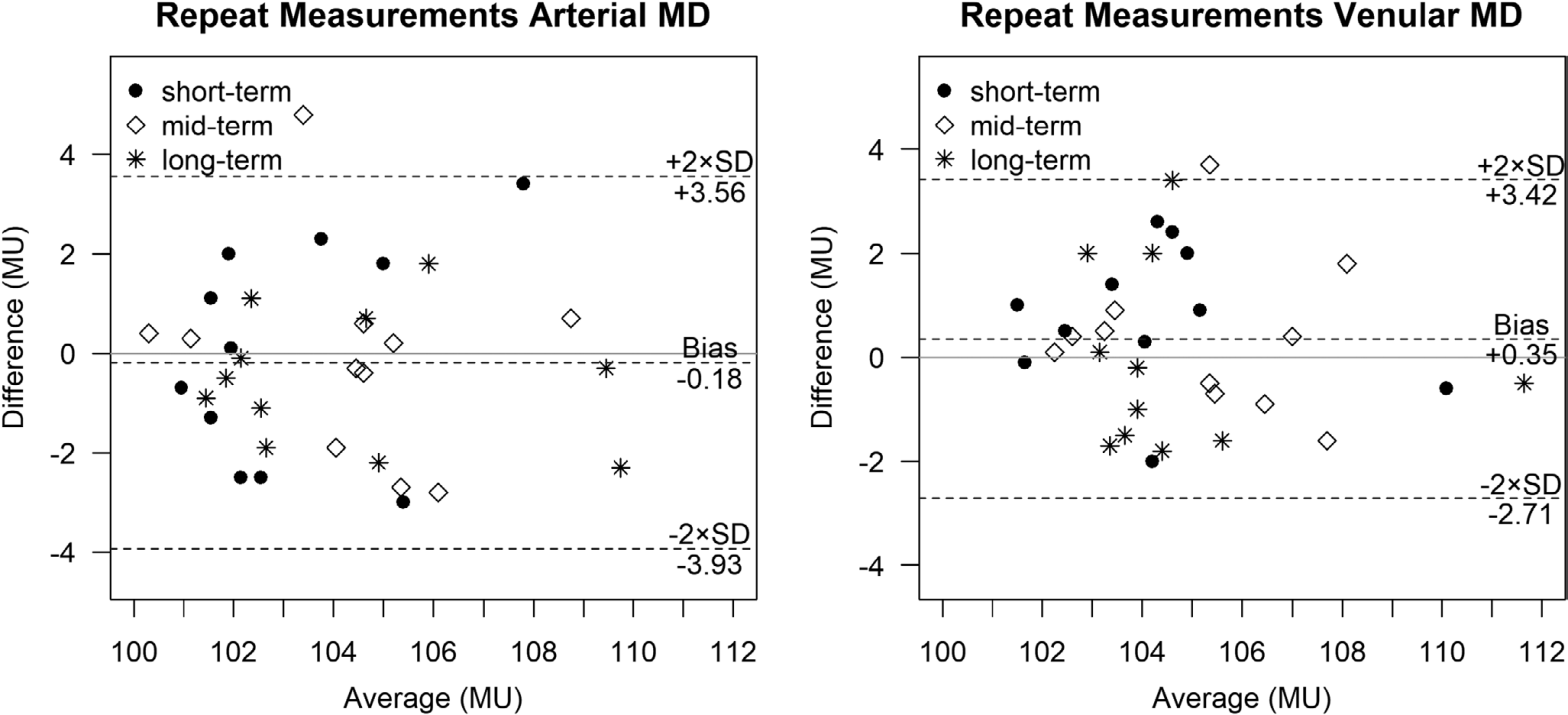
Bland–Altman plots of arterial maximum dilation (left) and venular maximum dilation (right) averaged across three flicker cycles (measuring units; MU) for short‐term (15–30 min), mid‐term (3–14 days) and long‐term (16–28 days) repeat measurements (*n* = 11, each).

All continuous variables were normally distributed, so parametric tests were employed. One‐way analysis of variance between groups (a), (b) and (c) revealed no statistically significant differences either for both measurement sessions (M1, M2) or for their difference (M1‐M2) (Table S1). Bland–Altman plots of all parameters (BDF, MD, MC, DA, BFR, RT, CT, *A*_max_, *A*_min_, *A*_peak_ and *V*_max_) using labelled data points belonging to all three subgroups of short‐term, mid‐term and long‐term repeats (a, b and c, respectively) did not reveal any significant systematic difference (bias) or trend of increasing variance with increasing time‐frame or otherwise (plots shown for arterial and venular Maximum Dilation only, Figure 3). Hence, ICC values were calculated including all 33 participants (Table 2).

**TABLE 2:** Mean ± SD (min–max) values of all outcome variables and intraclass correlation coefficients (95% confidence intervals) between measurements averaged across three flicker cycles (*n* = 33). Paired‐samples *t*‐tests were performed.

Parameter	Measurement 1	Measurement 2	*p* value	ICC (95% CI)
Arteries (*n* = 33)				
Baseline diameter fluctuation (%)	4 ± 2 (1.3–9.7)	3.9 ± 1.8 (1.3–8.4)	0.784	0.53 (0.22–0.74) moderate
Maximum dilation (%)	3.8 ± 2.6 (0.5–9.5)	4 ± 2.6 (0.1–10.9)	0.574	0.75 (0.56–0.87) good
Maximum constriction (%)	−3.4 ± 2.3 (−9.2–1.7)	−3.3 ± 1.7 (−6.2–1.6)	0.628	0.59 (0.31–0.77) moderate
Dilation amplitude (%)	7.2 ± 3.1 (1.8–13.1)	7.2 ± 2.9 (2.8–13.4)	0.955	0.69 (0.45–0.83) moderate
Baseline‐corrected flicker response (%)	3.2 ± 1.6 (0.2–7.8)	3.3 ± 1.7 (0.5–6.3)	0.596	0.70 (0.48–0.84) moderate
Reaction time (s)	16 ± 5 (8–29)	17 ± 5 (7–26)	0.782	−0.03 (−0.38–0.31) poor
Constriction time (s)	36 ± 7 (13–46)	35 ± 10 (0–47)	0.574	0.34 (0–0.61) poor
Veins (*n* = 33)				
Baseline diameter fluctuation (%)	3.1 ± 1.7 (1.1–8.9)	2.8 ± 1.6 (1.1–8.8)	0.203	0.69 (0.46–0.83) moderate
Maximum dilation (%)	4.8 ± 2.2 (1.6–11.4)	4.5 ± 2.4 (1–11.9)	0.193	0.79 (0.61–0.89) good
Reaction time (s)	22 ± 5 (15–35)	20 ± 5 (11–33)	0.155	0.05 (−0.28–0.37) poor

For both arteries and veins, Maximum Dilation was the most repeatable parameter with an ICC estimate of 0.75 and 0.79 (95% confidence intervals ranging from ‘moderate’ to ‘good’ according to the classification suggested by (Koo & Li, 2016)), respectively. Venular BDF showed lower levels of repeatability (0.69) and was more repeatable compared to its arterial counterpart (0.53). Reaction Time and Constriction Time for arteries and Reaction Time for veins were found to be the least repeatable (<0.34). Paired‐samples *t*‐tests showed no statistically significant differences between the two repeat measurements. Repeatability of RVA software‐generated responses (*A*_max_, *A*_min_, *A*_peak_ and *V*_max_) was on average worse than their counterparts generated using raw data analysis (arterial MD, MC, DA and venular MD, respectively) (Table 3). Paired‐samples *t*‐tests also revealed statistically significant differences for MC, DA (for both M1, M2) and venular MD (for M1 only) (Table 3).

**TABLE 3:** Intraclass correlation coefficients, across measurement sessions and comparison between software‐generated output (*A*_max_, *A*_min_, *A*_peak_, *V*_max_) and raw output flicker analysis (maximum dilation [MD], maximum constriction [MC], dilation amplitude [DA]), within measurement sessions.

Parameter (%)	Measurement 1	*p* value	Measurement 2	*p* value	ICC (95% CI)
*A*_max_	3.3 ± 2.1 (0–9)	0.329	3.1 ± 2.3 (−0.8–8.7)	0.109	0.72 (0.50–0.85) moderate
Maximum dilation (arteries)	3.8 ± 2.6 (0.5–9.5)		4 ± 2.6 (0.1–10.9)		0.75 (0.56–0.87) good
*A*_min_	−0.8 ± 1.1 (−3.6–2.1)	**<0.001**	−0.8 ± 0.9 (−2.6–1.2)	**<0.001**	0.05 (0.00–0.38) poor
Maximum constriction	−3.4 ± 2.3 (−9.2–1.7)		−3.3 ± 1.7 (−6.2–1.6)		0.59 (0.31–0.77) moderate
*A*_peak_	4.1 ± 2.6 (−0.6–10.8)	**<0.001**	3.9 ± 2.4 (−0.3–9.7)	**<0.001**	0.55 (0.26–0.75) moderate
Dilation amplitude (arteries)	7.2 ± 3.1 (1.8–13.1)		7.2 ± 2.9 (2.8–13.4)		0.69 (0.45–0.83) moderate
*V*_max_	3.6 ± 2.1 (0.6–9.3)	**0.046**	3.7 ± 2.7 (−0.5–10.1)	0.297	<0.00 poor
Maximum dilation (veins)	4.8 ± 2.2 (1.6–11.4)		4.5 ± 2.4 (1–11.9)		0.79 (0.61–0.89) good

*Note*: Values are expressed as means ± SD (min–max). Paired‐samples *t*‐tests were performed. Statistical significance is denoted in bold.

## 4 DISCUSSION

We have shown that, in general, superior repeatability is obtained for retinal vessel parameters computed from the raw arterial or venule diameter profiles in response to flicker light stimulation than the parameterisations provided by RVA software.

Substantial repeatability is paramount for any parameter should it be used for diagnostic, follow‐up, monitoring or risk stratification purposes. Despite the RVA being in the research forefront for over two decades and a number of reviews (Heitmar & Summers, 2012; Lim et al., 2013; Streese et al., 2020) and one consensus report (Garhöfer et al., 2010) being published highlighting shortcomings and possibilities, very little attention has gone into the assessment of flicker responses' repeatability.

In addition to the RVA software output, numerous groups have introduced a plethora of dilatory‐response parameters for arteries and veins (Heitmar & Summers, 2012; Streese et al., 2021). This proliferation in parameters is most likely due to the lack of a standardised parameterisation among research groups and the selective reporting of markers most suited to show differences in vessel responses in a diverse range of clinical groups with systemic (i.e. diabetes, cardiovascular disease) and ocular vascular disease (i.e. diabetic retinopathy, glaucoma) (Gugleta et al., 2012; Mandecka et al., 2007). Stratification between different pathologies has not been assessed widely, with only a few studies showing differences between disease stages (Mandecka et al., 2007) and systemic disease(s) (Al‐Fiadh et al., 2014; Günthner et al., 2022; Heitmar et al., 2017). Disease progression monitoring also requires good repeatability, as parameters with large variability could potentially mask disease progression. Unfortunately, most vessel parameters lack validation, at least in terms of repeatability.

The purpose of this study was to examine the repeatability of the vessel dilatory‐response parameters using the most commonly applied 3‐flicker (12.5 Hz) cycle protocol (Nagel et al., 2004) in healthy eyes. Although some data have been published on the repeatability of flicker responses, these are limited to a single parameter (Maximum Dilation) (Al‐Fiadh et al., 2014; Nagel et al., 2006; Noonan et al., 2013) or have become redundant with the introduction of the 12.5 Hz inbuilt flicker module (Garhöfer et al., 2003; Polak et al., 2000). Others have included repeatability data including responses with only one valid flicker cycle (Nguyen et al., 2009), whereas the main purpose of a repeat provocation protocol (three flicker cycles) is to minimise noise and variance and thus increase accuracy and confidence. So, when assessing repeatability, it is crucial to ensure all provocation cycles are completed and included in any subsequent analysis.

**FIGURE 4:**
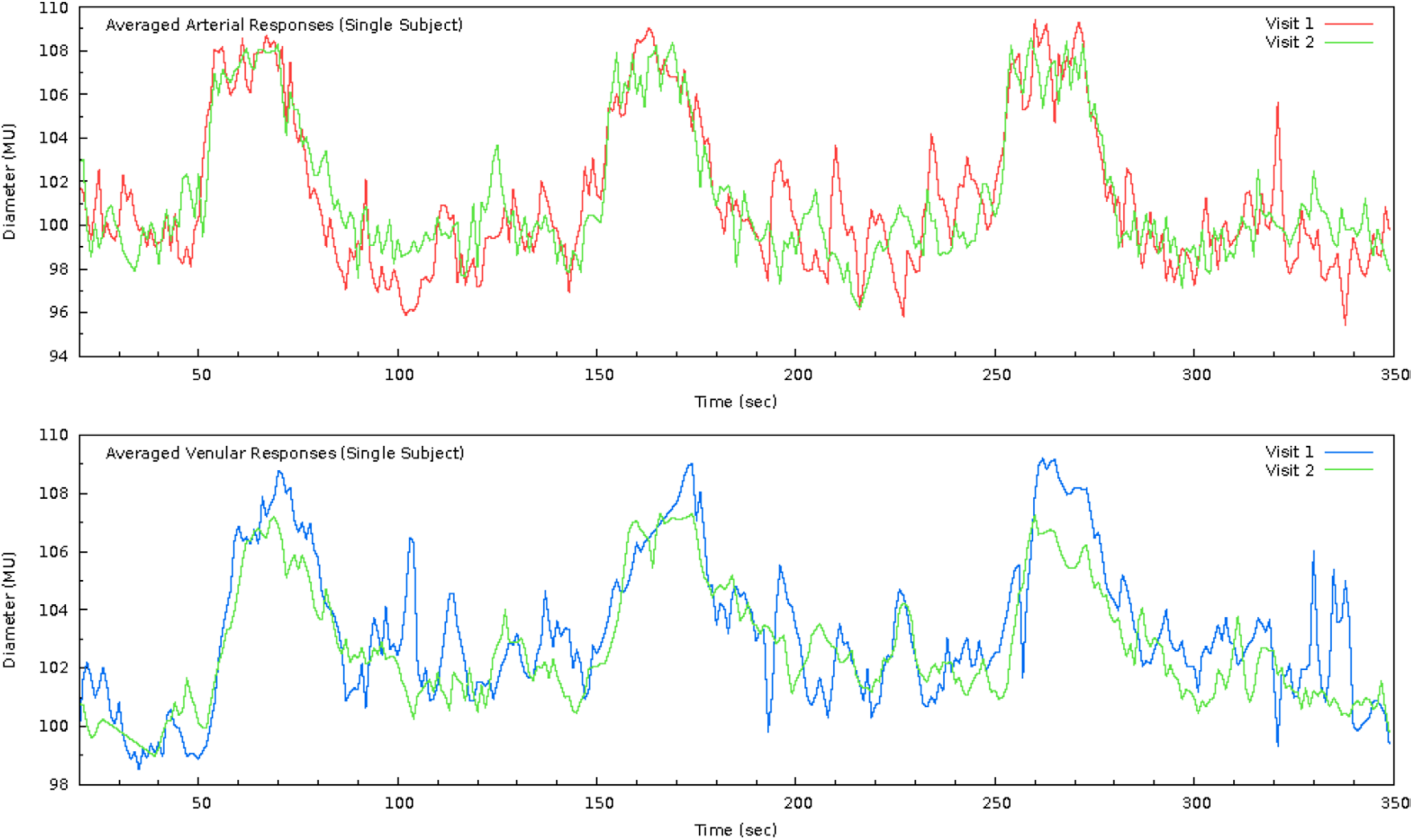
Sample flicker responses (raw data) for a single individual for arteries (top) and veins (bottom) for two measurement sessions 7 days apart (mid‐term). Initial 20 s have been discarded.

By adhering to a strict protocol where across measurements the location and vessel length were closely matched and only those measurements with three full flicker cycles were included, our analyses yielded ‘moderate’ to ‘good’ agreement for the majority of parameters, apart from blood vessel reaction times, which had ‘poor’ agreement (Koo & Li, 2016). A possible explanation for this could be due to the way Reaction Time and Constriction Time are calculated: the time taken from flicker start to the mathematical maximum/minimum (respectively) following flicker provocation. As most healthy individuals show a two‐stage reaction during flicker light provocation (Heitmar & Summers, 2015; Summers & Heitmar, 2025) characterised by a rapid diameter increase followed by a plateau, the time until the mathematical maximum can vary substantially depending on the extent of the plateau phase (Figure 4). Similarly for arterial constriction and Constriction Time.

Software‐generated parameters appear to underestimate vessel responses in both arteries and veins when compared to the independently analysed values (Table 3), in line with previous findings (Heitmar et al., 2010; Streese et al., 2021). This is a direct consequence of the entirely dissimilar definitions of the two: the former are calculated as the mathematical average across a narrow time window between 17 and 23 s after flicker initiation, whereas the latter select the mathematical maximum (or minimum) within a wider time window post flicker start. The assumption of the definition of the software‐generated parameters is that the range of higher (or lower) dilatory responses expected to be found within 3 s prior to flicker cessation and 3 s after is proving problematic, as most young individuals reach their maximum much earlier than this 6‐s window (Heitmar et al., 2010; Heitmar & Summers, 2015; Streese et al., 2021; Summers & Heitmar, 2025). Constriction Time, for instance, in our sample population is found to fluctuate around 36 ± 7 (for M1) and 35 ± 10 (for M2) seconds, which includes time points well beyond that narrow 6‐s range (Table 2). Hence, the differences for *A*_min_ and *A*_peak_ to their counterparts (MC and DA, respectively) are found to be significantly different. Similarly, albeit to a lesser extent, this is true for venular RT values; hence, the differences are borderline significant for M1 and insignificant for M2. Arterial MD is not statistically significantly different from *A*_max_, but the trend still exists. The strength of this study lies within the standardised approach to data collection and analysis, in particular, including only data where all three flicker cycles were completed and by keeping vessel lengths constant. Additionally, the vast majority (18/22) of participants had repeat tests at the same time of the day (for mid‐ and long‐term visits) to mitigate the effect of potential diurnal variations. While, on the other hand, one of our limitations is the relatively small sample size and the fact that we examined a multi‐ethnic cohort. However, as most of our clinical cohorts consist of multi‐ethnic groups, it was important for us to reflect this in our sampling.

In conclusion, our data on repeatability highlights the importance to consider the strength of some parameters vs. others when choosing a suitable parameter identifying group differences and to plan follow‐up measurements. Our results are in agreement with the sample published in healthy Asians (Nguyen et al., 2009) but highlight the variability of the parameters derived from retinal vessels during flicker provocation. Future studies should consider the data spread of retinal vessel reactivity parameters when considering effect size and sample size calculations.

## Supporting information


Table S1:
