# Supplementary material for: Repeatability of retinal vessel flicker responses in healthy individuals
Source: Acta Ophthalmol. 2025 Aug 12;104(2):e165–72. doi: 10.1111/aos.17578 (PMC12888953; doi:10.1111/aos.17578)
Supplement: Supplementary file 1 — Table S1: [file AOS-104-e165-s001.docx]

| **Parameter** | **One-way ANOVA between groups (a), (b) and (c) (n=11, each)** | | |
| --- | --- | --- | --- |
| **Arteries** | **M1** | **M2** | **M1-M2** |
| Baseline Diameter Fluctuation (%) | F(2,30) = 1.166, p = 0.325 | F(2,30) = 2.493, p = 0.100 | F(2,30) = 1.231, p = 0.306 |
| Maximum Dilation (%) | F(2,30) = 0.558. p = 0.578 | F(2,30) = 1.022, p = 0.372 | F(2,30) = 0.270, p = 0.765 |
| Maximum Constriction (%) | F(2,30) = 0.371, p = 0.693 | F(2,30) = 0.223, p = 0.802 | F(2,30) = 1.148, p = 0.331 |
| Dilation Amplitude (%) | F(2,30) = 0.731, p = 0.490 | F(2,30) = 1.423, p = 0.257 | F(2,30) = 1.446, p = 0.251 |
| Baseline corrected Flicker Response (%) | F(2,30) = 0.946, p = 0.400 | F(2,30) = 1.137, p = 0.334 | F(2,30) = 1.531, p = 0.233 |
| Reaction Time (s) | F(2,30) = 1.361, p = 0.272 | F(2,30) = 0.769, p = 0.472 | F(2,30) = 2.041, p = 0.148 |
| Constriction Time (s) | F(2,30) = 2.445, p = 0.104 | F(2,30) = 0.385, p = 0.683 | F(2,30) = 1.108, p = 0.343 |
| **Veins** |  | | |
| Baseline Diameter Fluctuation (%) | F(2,30) = 1.338, p = 0.278 | F(2,30) = 0.824, p = 0.448 | F(2,30) = 0.164, p = 0.849 |
| Maximum Dilation (%) | F(2,30) = 0.390, p = 0.681 | F(2,30) = 0.655, p = 0.527 | F(2,30) = 0.811, p = 0.454 |
| Reaction Time (s) | F(2,30) = 0.306, p = 0.739 | F(2,30) = 0.057, p = 0.945 | F(2,30) = 0.058, p = 0.944 |

**Supplemental Table 1**: Comparison between (a) short-term (15-30 minutes), (b) mid-term (3-14 days) and (c) long-term (16-28 days) repeat measurements (M1, M2) and their difference (M1-M2) for all arterial and venular parameters averaged across three flicker cycles.
